# Preparing for Mental Health Act reform: Pilot study of co-produced implementation strategies for Advance Choice Documents

**DOI:** 10.12688/wellcomeopenres.17947.1

**Published:** 2022-07-07

**Authors:** Lucy A. Stephenson, Tania Gergel, Alex Ruck Keene, Larry Rifkin, Gareth Owen

**Affiliations:** 1 Institute of Psychiatry, Psychology & Neuroscience, King's College London, London, SE5 8AB, UK; 2 39 Essex Chambers, 81 Chancery Lane, London, WC2A 1DD, UK; 3 Dickson Poon School of Law, Kings College London, London, WC2R 2LS, UK; 4 South London and Maudsley NHS Foundation Trust, London, UK

**Keywords:** Advance directive, advance choice document, co-production, implementation, quality improvement, mental health act, mental capacity, bipolar

## Abstract

Background

Advance Decision Making (ADM) is strongly supported by stakeholders but implementation remains challenging. In England and Wales, implementation strategies are urgently required to prepare for the introduction of mental health ‘Advance Choice Documents’ (ACDs) as part of Mental Health Act reforms. We report on a pilot project which aimed to co-produce and evaluate implementation strategies for ACDs with those who experience fluctuating mental capacity in the context of bipolar.

Methods

A co-produced prototype ACD template was piloted in ‘Plan, Do, Study Act’ (PDSA) cycles. Implementation strategies were co-produced with participants and mapped onto the Expert Recommendations for Implementing Change (ERIC) framework. Strategies were evaluated during thematically analysed qualitative interviews.

Results

We piloted the template with 17 service users during 5 successive PDSA cycles and conducted 75 in depth interviews with stakeholders. Key strategies identified as accessible, appropriate and feasible were: interactive assistance from an independent ‘supporter’, a structured template and active offers of involvement to service users and informal carers.

Conclusions

Mental health professionals and organisations must prepare for increased expectations around mental health ADM. We recommend further pilot projects and the establishment of ‘ACD workshops’. Resource is essential to fund independent ‘supporters’, training, network building and embedding ADM in clinical pathways.

## Introduction

Mental health advance decision making (ADM) is about supporting service users who live with severe mental illness (SMI) to make use of their expertise and state preferences for treatment in any future mental health crises. ADM is particularly salient for those with fluctuating decision-making capacity for treatment (DMC-T) and are at risk of experiencing compulsory treatment. A paradigm example is the experience of people who live with Bipolar Affective Disorder (bipolar). When well, individuals are very able to engage in decisions about their treatment. However, during relapses, particularly manic phases, DMC-T may be rapidly lost then re-gained during the recovery period ( Owen *et al*., 2008). Research suggests that there is both an ethical and practical imperative for statutory ADM ( Owen *et al*., 2019). Ethical reasons include respect for service user interest ( Hindley *et al.*, 2019; Swanson *et al.,* 2006b), autonomy and reducing inequalities in decision making (Council of Europe: Enabling citizens to plan for incapacity) . Practical reasons include potential therapeutic benefit ( Stephenson *et al.,* 2020b) and reduction in the need for compulsory treatment ( de Jong *et al.*, 2016; Molyneaux *et al.*, 2019). Multiple jurisdictions (including USA, India, Australia, Netherlands and Scotland) have now introduced tailored, statutory support for ADM. However, in jurisdictions where outcomes have been monitored uptake of mental health ADM has remained low ( Hindley *et al.,* 2019; Swanson *et al.,* 2006b Mental Welfare Commission for Scotland: Advance statements in Scotland).

Research in this field has already identified multi-level barriers to implementation which need to be overcome. Shields *et al.* ( Shields *et al.,* 2014) conducted a systematic review of barriers and results included: concerns about legal provisions and liability, resource implications, lack of knowledge amongst health professionals and service users, concerns about clinical feasibility including mental capacity, lack of support for service users and concerns that documents would not be accessed or applied. The scale of these barriers are unsurprising given that according to Medical Research Council guidance ADM can be understood as a complex intervention; it requires multiple components and various behaviours at several points in time from multiple groups ( Skivington *et al.*, 2021). Some implementation strategies have been suggested around engaging and supporting stakeholders and addressing practical issues ( Stephenson *et al.,* 2020b; Zelle *et al.,* 2015). But so far, these strategies have not been co-produced with stakeholders, piloted or held within a comprehensive implementation science framework which would facilitate wider evaluation and use.

In England, the setting for this project, grappling with ADM implementation is an urgent issue as government has committed to introducing ADM documents in the form of ‘Advance Choice Documents’ (ACDs) (Reforming the Mental Health Act: Department of Health and Social Care). At present the only legal provision for ADM is under the Mental Capacity Act 2005 (MCA) for ‘Advance Statements’ and ‘Advance Decisions to Refuse Treatment’. These provisions do allow service users to state treatment requests and refusals for mental health treatments, but they do not have formal legal status if the person is detained under the Mental Health Act 1983 (MHA). In contrast, according to a recent White Paper (Reforming the Mental Health Act Government Response) ACDs would retain some legal weight if the person were detained under the MHA. These reforms are likely to impact a significant proportion of service users given that in 2019–2020 alone 50,893 new detentions under the MHA were recorded (Mental Health Act statistics annual figures 2019–2020). However, in order to ensure that these reforms achieve their intended goals anticipatory research focussed on implementation is urgently required.

Therefore the aims of this study were to:

1. Co-produce, describe and pilot implementation strategies for ACDs with key stakeholders
2. Evaluate the acceptability, adoption, appropriateness and feasibility of these implementation strategies
3. Recommend implementation strategies for future pilots and wider roll out

## Methods

### Study design

Implementation experts draw an important distinction between an intervention, ‘the thing’, and implementation strategies, ‘the stuff that helps you do the thing’ ( Curran, 2020). For clarity, in this study the intervention was formal mental health ADM which relies on the existing legal framework of the Mental Capacity Act 2005 (MCA) provision for Advance Statements and Advance Decisions to Refuse Treatment. We could not use actual ACDs as the intervention as the reforms have not yet been passed but shaped the template to mirror the shape of anticipated reforms as closely as possible. Participants were made aware of the current limitations of the legal framework. As the intervention has already been established the key outstanding questions for service users, their family members, clinicians and mental health organisations are around which implementation strategies could help utilise ACDs. 

To address our first aim service user participants along with a supporting family member/friend and a treating health care professional were invited to take part in creating a prototype ACD using a collaborative template called a ‘Crisis PACk’ (available in a data repository). This template was based on a document co-produced with stakeholders during a previous study ( Stephenson *et al.*, 2020b) and adapted to the needs of the local setting. Of note, the template also offers service users the option to ‘self-bind’ i.e. to request compulsory treatment in advance even if they anticipate that when they are unwell and the treatment is needed they are likely to refuse it. This option was included in response to evidence that it may have particular salience for people with bipolar ( Gergel *et al.,* 2021; Gergel & Owen, 2015; Stephenson *et al.,* 2020a)

Participants created their documents in successive ‘Plan, Do, Study Act’ cycles according to ‘Model for Improvement’ methodology ( NHS Online library of Quality Service Improvement and Redesign tools; Langley *et al.*, 2009). This methodology was selected as it is supported by the site of implementation and allows for rapid feedback and involvement from stakeholders. Throughout the process stakeholders were asked to generate ideas for implementation strategies, including developing the template itself. This information could then be applied for further refinement and feedback in the next PDSA cycle. Informal feedback was collected from all participants throughout the process with prompts in all contacts e.g. administrative phone calls and emails with an open invitation for participants to email the project lead.

To address our second aim, in depth qualitative interviews were conducted with participants from all stakeholder groups before and after they had completed their documents to explore their experiences. Interviews were undertaken by a clinical researcher (L.A.S, female, white) who has had over 10 years clinical experience working in south east London and training in psychiatry and psychotherapy. The participants were not known to the researcher prior to the project but relationships were established with participants over the study course during repeat interviews and contacts. The participant information sheet contained information about the researcher’s roles and aims for the project. Throughout the project topic guide domains were updated to reflect new implementation strategies and ask for feedback on them. A measurement plan was created to govern the collection of quantitative data about the process of making the document.

The refined implementation strategies were described according to criteria outlined by Proctor *et al.* ( Proctor *et al.*, 2011) and mapped onto the Expert Recommendations for Implementing Change (ERIC) framework ( Powell *et al.*, 2015).

### Participants and procedures

The project took place between January 2020 and November 2021. We targeted English speaking service users with a primary diagnosis of bipolar documented by a health professional. Records were reviewed by a clinical researcher (L.A.S) to ensure that the diagnosis was consistent with their recorded presentations. Participants had to have experienced at least 1 detention under the MHA, be interested in making an ADM document and have capacity to make an ADM document. Participants were all in active contact with secondary mental health services in South London and Maudsley NHS Foundation Trust (SLaM) for treatment of bipolar. SLaM is a busy, large, urban mental health trust offering a range of services to a diverse population in south-east London. Participants were recruited from a variety of settings across the trust: inpatient wards, Home Treatment Teams and Community Mental Health Teams. Participants self-referred to the project or a treating clinician suggested it. Once participants had been referred to the project they were contacted by phone or email to discuss what the project would involve and sent written materials with further details.

Once the service user participant had been recruited, we asked permission to interview a health professional and a carer/family member/friend/advocate who would be involved in making their document. We set out to recruit 12 service user participants, 12 family/friend participants and 12 health professional participants to make ADM documents and participate in the qualitative interviews. This sample size was chosen as sufficient to achieve qualitative data saturation whilst being manageable.

***Data collection.*** Interviews were held online/by telephone (depending on participant preference and access to technology) due to pandemic restrictions. Participants were informed interviews would last approximately 1 hour but length was guided by the participants and according to their level of comfort and time restrictions (e.g. many clinicians were time limited due to providing front line services during pandemic). Interviews were semi-structured using a pre-prepared topic guide. Question domains were informed by a previous survey ( Hindley *et al.*, 2019) and focus group study ( Stephenson *et al.*, 2020b). The topic guide was discussed amongst the research group and Service User Advisory Group. It was adapted throughout the project to ensure the content was driven by participant experience and to probe emerging themes. Interviews were conducted with all participants before and after a document had been made. Arrangements were made to follow participants up and conduct a final interview after any crisis and/or at the end of the study period. The results of the final time point interviews will be reported in a forth coming publication. Following the interview participants were advised that they could contact the researcher at any point if they wished to make additional points or change their expressed views. There was a team consensus that data saturation was achieved within the sample of interviews.

### Consent

The process of consent was impacted by the pandemic which prevented face-to-face contact. All participants in the qualitative interview study were sent the participant information sheet and consent form in advance by email or post according to their preference. A remote contact was then arranged by a clinical researcher to read through the information sheet and consent form point by point. Where handwritten consent was not possible participants were asked to confirm consent via email from their personal account. Consent was also confirmed verbally before and after each qualitative interview. For those participants who did not take part in the qualitative interview research study but still wished to make an ADM document written consent was not required as this part of the project fell under the governance of the mental health trust Quality Improvement team. This did not require formal Research Ethics Committee approval as ADM is already supported in the MCA. It is not a novel intervention and should already be part of standard clinical care for bipolar as recommended in NICE guidelines ( National Institute for Health and Care Excellence, 2014). These participants were given written and verbal information about the project and their consent to take part confirmed verbally by a member of the project team and/or their treating clinician at each contact.

### Ethics

Ethical approval for the qualitative interview research study was granted by Camberwell and St Giles ethics committee (REC reference 19/LO/1142). Initial approval was granted on 03.09.2019 with an amendment approved on 04.08.2020 to allow the research to continue under pandemic restrictions. The part of the project which involved the Quality Improvement process (i.e. making the ADM document) was approved and supported by the SLAM Quality Improvement team. This process included project review, training and support from a Quality Improvement mentor.

### Analysis

Interviews were audio recorded, transcribed and uploaded to a data management programme (NVivo Release 1.5 (4577). Data was thematically analysed following Braun and Clark ( Braun & Clarke, 2006) during a process which included regular, reflective interdisciplinary team meetings. The team included experts in psychiatry (GO, LR, L.A.S), philosophy (TG), lived experience (TG) and law (ARK). This diverse interdisciplinary environment supported a reflective, rounded process of data analysis. One researcher (L.A.S) read all the transcripts with two other researchers (GO and TG) read a sample. Initial ideas were noted and then discussed to generate an initial coding tree. L.A.S then coded all transcripts with GO and TG coding a sample, discrepancies were discussed and a final coding tree agreed. Proposals for emerging themes were discussed at team meetings then checked against the data for coherence. The overarching thematic analysis of participants experience and methodology will be presented in a forthcoming publication. In the present publication we report on specific feedback around implementation strategies. Standard descriptive statistics were used to summarise and analyse quantitative measures.

## Results

### Characteristics

Overall, 36 service user participants were referred to the project. Of this group 23 (64%) accepted the initial offer of making a document and in total 17 people (47%) drafted documents. Reasons for not moving onto make a document are outlined in Table 1. Of the 17 service users making documents 14 were eligible and wished to take part in the qualitative interview study, this involved completing a demographics questionnaire. The demographics of these participants are outlined in Table 2– Table 4. Of those who drafted documents but did not complete them 1 was not completed due to pandemic interruption, 1 required more time due to a health professional absence, 1 preferred to not include health professionals in the process and 1 required more time as the service user experienced relapse. No participants expressed a wish to drop out of the process completely. 

**Table 1.** Reasons for declining to make a Crisis Pack.

Reason	n
No longer in secondary care services	1
Unable to contact	7
Team members not supportive	3
Too unwell	3
Other commitments	1
Concerns stress of process may trigger relapse	2
Reason not stated	2

**Table 2.** Service user participant demographics

Service user participants	n=14
**Age (years; mean (SD; range))**	36.6 (12.4; 23-58)
**Gender (female/male)**	8/6
**Ethnicity**	
Black	6
White British	6
White Other	2
**Relationship status**	
Not in a relationship	10
In a relationship	4
**Highest Level of Education**	
GCSE	2
A-Level	3
University educated	9
**Employment**	
Unemployed	7
Employed	6
Student	1
**Benefits**	9
**Diagnosis**	
Bipolar 1	14
**Number of times hospitalised**	
0	0
1-5	7
5-10	5
>10	2
**Number of times detained under MHA**	
0	0
1-5	8
5-10	4
>10	2
**Services currently involved**	
General Practitioner	8
Community Mental Health Team	12
Specialist service	3
Home Treatment Team	1

**Table 3.** Family/friend participant demographics.

**Friends and family participants**	n=14
**Age (years; mean (SD; range))**	45.46 (17; 19–74)
**Gender (female/male)**	9/5
**Ethnicity**	
Black	6
White British	6
White Other	2
**Relationship with loved one**	
Parent	6
Sibling	2
Partner	3
Child	2
Friend	1
**Personal use of services**	
Mental Health	0
Physical Health	3
Not using services	9
Prefer not to say	2

**Table 4.** Health Professional Participants.

Health Professional Participants	n=18
**Years of professional experience** ** following primary qualification (mean** ** (SD; range))**	14.06 (11.5; 1.5–40)
**Gender (female/male)**	9/9
**Ethnicity**	
Black	4
White British	8
White Other	2
Asian-Indian	3
Prefer not to say	1
**Primary professional role**	
Psychiatrist	6
Psychologist	2
Care Coordinator (nurse)	6
Care Coordinator (social worker)	2
Care Coordinator (psychologist)	1
Advocate	1
**Clinical setting**	
Community Mental Health Team	14
Early Intervention Team	2
Home Treatment Team	1
Advocacy service	1

### Outcomes

The outcomes and process measures of the intervention strategies employed to support all participants in making their ADM document are detailed in Table 5.

**Table 5.** Quantitative outcome and process measures.

Stage of process of making Crisis PACk	Action being measured	Result
Identifying	Number of documents offered	36
	Number of documents accepted	23
	Service User identifies need	4
	Health Professional identifies need	32
	Family member/Friend identifies need	0
	Identified by Community Mental Health Team	21
	Identified by Home Treatment Team	5
	Identified by Early Intervention	2
	Identified by inpatient	4
Drafting	Number of documents drafted	17
	Drafted on paper	4
	Drafted on personal computer	3
	Supported to draft on trust computer	10
	Support from Care Coordinator	1
	Support from Crisis PACk team member	10
	Support from family member	2
	Support from Care Coordinator and Crisis PACk team member	2
	No support	2
	Average amount of support time required (minutes)	38
Discussing	Number of documents ready to be discussed	16
	Number of documents completed	13
	Number of discussions involving Consultant Psychiatrist	12
	Number of discussions involving Care Coordinator	11
	Number of discussions involving psychologist	2
	Capacity confirmed by Care Coordinator	2
	Capacity confirmed by Psychiatrist	13
	Average number of meetings per Crisis PACk	1
	Average length of meeting (minutes)	78

### Co-producing and evaluating implementation strategies

Implementation strategies were generated and refined throughout the study period in 5 successive PDSA cycles. These PDSA cycles are summarised in Table 6, and the final implementation strategies are summarised in Table 7. This table contains the strategies used by the research team to facilitate organisational engagement as well as co-produced strategies generated and evaluated by project participants. The table maps the strategies co-produced by the project participants and the interdisciplinary research team onto the ERIC ( Powell *et al.*, 2015) arranged into concept mapped clusters for easier references ( Waltz *et al.*, 2015). Below, the refined implementation strategies are described according to their ERIC cluster.

**Table 6.** PDSA cycle learning summary.

	PDSA Cycle 1		PDSA Cycle 2	PDSA Cycle 3	PDSA Cycle 4	PDSA Cycle 5
Key events	Preparing for PACT implementation	On pause during pandemic	Converting PACT to Crisis PACk	Implementing version 1 of Crisis PACk	Implementing version 2 of Crisis PACk and refined process	Implementing version 3 of Crisis PACk and refined process
Dates	November 2019 – February2020	February 2020- June2020	June 2020- August 2020	August 2020 – January 2021	January 2021-February 2021	February 2021 – August 2021
Key issues identified	Template co-produced through series of stakeholder focus groups and consultation process Detailed written feedback on starting template from 2x SU, 1xFF, 2xHP Overall positive feedback about format, layout and language Need for clarification on summary page Need for clarification on sections around MHA/MCA assessment – tension around the audience for the prompts on these boxes – whether for SU or HP, some confusion about whether they were distinct assessment or overlapping. Concern that service users would not understand what ‘capacity’ meant. Mixed feedback about whether to keep prompts on form or transfer to guidance documents Recommendations for collapsing sections to condense the form. Noted complexities around finding right pace of information giving for all participants.	**Language difficulties:** Advance Care Planning/ Advance Decision Making/Advance Directive Crisis PACk (Preferences for Admission, treatment and Care) decided upon as meaningful term, in keeping with ‘patient journey’ ethos and allowed for flexible nature of the document. **Increased** ** importance of ADM** ** in pandemic:** Concern expressed that the destabilising nature of the pandemic may make ADM an even more urgent task for SUs. Concerns about the need for awareness and respect of physical health ADRT during the pandemic	**Physical health advance** ** decision making** Extent to which physical health and end of life care planning should be included. Concern raised that it may not be appropriate to ask younger (or vulnerable) service users about preferences for refusing active treatment **Endorsement** Formatting endorsement sections Language around representing appropriate balance of power Concern that this section may be overly legalistic and more about reducing potential liability for clinician than representing service user views Appropriate prompts to guide conversations Practical difficulty of including signatures **Capacity** Discussed whether to include a capacity assessment. Concern that although service users who do not have DMC-T should still be able to make the document capacity assessment should be encouraged. Concern that retrospective capacity assessments are extremely difficult and could undermine the document. Need for flexibility – service users could choose to have capacity assessment or not Careful language required around framing assessment **Synchronising with current** ** SLAM systems** Aim to create an online version to go in electronic records. This would link to BETH a service user interface	**Document format and** ** flow** Noted feedback that more clarity required on who fills out which section and when in the process. **Feedback from lead** ** nurse for reducing ** **restrictive practice** ** incorporated** We requested and received feedback on prompts around preferences for inpatient admission. These included planning for trauma informed restrictive practice. **Digital poverty** Two participants did not have access to a computer with internet. It was noted that the process should be feasible for those living in digital poverty. One of these service users was supported by a family member and one by a Care Coordinator and project team member to write up their documents	**Document format and flow:** Positive feedback on this, more intuitive to complete **Advantages of an ** online form:Additional detailed guidance could be available in hyperlinks Online form could significantly reduce work burden for dissemination e.g. by automatically generating alert, sending copy to GP, sending copy to LCR. **Guidance** **documents:** Noted that lengthy guidance documents rarely referred to	**Support required for drafting:** Noted support enabling for the majority for a variety of reasons; practical and emotional. **Support required for ** **discussion:** Confirmed suggested agenda to send out to meeting participants in advance and need for clinicians to actively ‘translate’ SU preferences into clinically feasible plans to maximise the chance that future colleagues will follow these plans **Need for training:** Clear that further training is required for clinicians to support these processes **Need for further** ** engagement with** ** liaison and bed ** **management teams** This is required to support application of document contents, increase confidence of crisis teams in using the document
Template adaptations	On balance the team decided that prompts should remain on the form.	Name change decided on: PACT to Crisis PACk Decision made to have a more flexible Word working document rather than fillable PDF	Box prompting service users to identify physical health concerns that may need to be managed in a mental health inpatient setting ‘Light touch’ on physical health care planning with plans for more detailed ‘modules’ for services where this is more relevant i.e. Older Adults Removed some of the declarations and all signatures. Replaced with boxes for names of those involved in the discussions Capacity assessment framed as ‘do you have any reason to doubt the service user’s capacity to make this document’. Document can be made with or without capacity ‘module’. Included an ‘Others views’ section to involve whole MDT Insertion of SLAM Crisis and Safety Plan to replace section on relapse indicators	Document colour coded and sections grouped according to roles in completing the section. E.g. SU sections coloured blue and grouped together at the beginning of the document to reflect the fact that the first stage is the SU drafting the document. A section on SU described thresholds for admission was inserted to reflect the naturally emerging participant discussions on this. The shared treatment recommendations were moved til the last page and functioned as a summary Inpatient preferences updated to prompt inclusion of information to support trauma informed care and managing first 72 hours of an admission when leave is often restricted.	Focused on providing very brief guidance for all parties on introductory page of the form with future space for hyperlinks to further guidance on an online form included.	Re-formatted preferences section into one table for briefer read at point of crisis Added ‘additional comments’ section as suggested by participant – provide blank space to maximise flexibility of the document
Process adaptations	Support required for HP and SU, helpful to have a 3rd party facilitator read prompts on form to enable SU and HP to focus on conversation around the content Considered Recovery College workshop as a ‘hook’ for HPs and SU Importance of identifying making the document as a significant and potentially demanding piece of work for SU.			Enquiry about values behind preferences to help clarify answers noted as useful in initial meetings	**Engaging** ** professionals** Observed that professionals seemed more likely to engage at service user request i.e. service user shown active interest and had started drafting with support of project team. **Timing of offer** Works well pre- discharge from community team to consolidate expertise **Support for** ** professionals** Emphasise need for active professional input in creating shared recommendations **Online meetings** Viable, screen sharing document works well to facilitate discussion	**Developed format for** ** an online ‘support ** **session’** involving arranging online meeting with SU, screen sharing the document and reading out form prompts. **Developed process** ** for coaching** ** professionals** Advance email outlining basic agenda sent to all. Met with professionals only prior to meeting starting to address questions, outline their main tasks (i.e. capacity assessment and involvement in shared recommendations), when SU and FF joined discussed chairing arrangements, suggested screen sharing document with ‘typing as we go’ then sent round to all parties after the meeting to review.
Crisis Pack Working Group Meetings		Crisis Pack Working Group formed – email contact between team remotely editing template	July 2020	August 2020	January 2021	February 2021

**Table 7.** Implementation strategies mapped onto relevant ERIC categories.

Strategy number	Strategy description	Actor	Action	Target of the action	Temporality	Dose	Implementation outcome affected	Justification
Use evaluative and iterative strategies								
46	Obtain and use service user and family feedback	Researcher	Formal feedback collected during in-depth qualitative interviews Informal feedback requested and collated from all stakeholder participants throughout project	Service users, family members and health professionals involved in making ADM documents	Interviews conducted before and after documents had been made	Aimed for interviews at 2 time points Participants invited to email project coordinator at any time with feedback	Acceptability Feasibility Appropriateness	To facilitate co-production and evaluation of implementation strategies
14	Conduct cyclical small tests of change	Researcher	Conducted successive PDSA cycles	Service user, family member/friend/health professional project participants	Throughout the pilot project	5 cycles	Acceptability Feasibility Appropriateness	Method supported by the mental health trust Quality Improvement team and a good methodological fit for piloting new implementation strategies for a pre-existing intervention in clinical practice
Provide interactive assistance								
33	Facilitation	Created role for a ‘supporter’	Supporter offers 1:1 session to draft their sections of the document. ACD supporter reads out prompts and available to answer any questions, can assist with typing service user answers (verbatim) into the form if required Supporter offered suggested meeting agenda and coaching/ guidance before and during meeting for health professionals Supporter actively offers to facilitate involving trusted family member/friend. This is achieved with outreach phone call/email and including consideration of their availability in coordinating meetings.	Overcome barriers around IT access and confidence, literacy issues. Provide emotional support, enable service user to confidently prepare for meeting which may help manage power imbalance ACD supporter offered suggested meeting agenda and coaching/guidance before and during meeting Family member/friend involved in making document. Increase confidence of family member/friend to contribute to document and be involved in meeting.	Offer of support to service user and health professional made after service user has signalled interest in making document	1 email to make contact with health professional Offer of informal coaching/trouble shooting by video call prior to meeting 1 email/phone call to make contact with family member/ friend 1–2 sessions to support service user in drafting document prior to meeting approx 30–60 minutes Offer attendance at meeting to discuss document and facilitation if preferred Offer of assistance disseminating document Offer support to trouble shoot with any queries	Acceptability Adoption Appropriateness Feasibility Fidelity	*‘I know some services like can just give patients the* * piece of paper with all the questions and ask them to fill * *them out… it was like the emotional aspect of it really, * *as well… going through it bit by bit as well was really* * helpful… And you know, getting ideas as well…(about) * *what other service users might say, within the care* * package, like it sparks another thing of what you* * would actually like to add as well.’ (Service User 10 Post * *Document Interview)* *‘I think it was really helpful that we worked through it * *before, on a call, because I wouldn’t have been able to* * think it out as thoroughly by myself, just because there’s* * just lots of questions… because it gives you time to like* * think…in a setting where it’s like, you know, the decision * *isn’t final yet so you can still think about it… I do feel* * that, you know, it is nice to have some time to think* * about your answers, and to go through it in a bit of an * *informal sort of way.’ (Service User 1 Baseline Interview)* *‘It’s the kind of thing that I probably wouldn’t give that * *much time to, because I don’t really want to be doing * *it. So it’s good for someone else to do it.’ (Service User 3 * *Post Document Interview)* *‘although it kind of takes a bit more resource, to kind * *of have a focus on the interpersonal atmosphere and * *connecting with the person, in the end it might actually* * become more efficient?’ (Health Professional for Service* * User 15 Post Document Interview)* *‘People should not be doing this project if their heart’s* * not in it, and there should be a specific role based* * interview for people to take on this (supporter) role…Its * *not for the fainthearted, it’s not just for people who* don’t give a f***’ (Service user 14 Post Document * Interview)* *‘I think it’s actually quite distinct, because there’s a focus* * on something, which wouldn’t necessarily be the focus if * *we had like a CPA* (Care Programme Approach *) review.* *’ Health Professional for Service User 7 Post Document * *Interview* *‘supported me the whole way…because it’s the first one* * I’ve done, I felt quite nervous, that I’d do it wrong…it * *was so helpful…it was fantastic actually, because all * *three of them, it was the first meeting we’d had' Health * *Professional for Service User 4 Post Document Interview* *‘it would be beneficial for it to be an independent * *person, personally….because I know some people still * *don’t have trust within their care co-ordinators or, as* * well, not even their care co-ordinators, just services in* * general.’ Service User 10 Post Document Interview’*
54	Provide local technical assistance							
53	Provide clinical supervision							
Adapt and tailor to context								
63	Tailor strategies	Interdisciplinary research team, senior SLAM clinicians, service user advisors	Formed working group to adapt PACT template to local needs Generation and adaption of process implementation strategies driven by feedback from participants in PDSA cycles	Adapting materials and strategies for local context	Regular working group meetings held while the pilot project was running	4 meetings in 12 months	Acceptability Adoption Appropriateness Feasibility	Necessary to coordinate efforts around ADM to prevent a split between the research project and clinical projects which would make both unfeasible.
51	Promote adaptability							
Develop stakeholder interrelationships								
35	Identify and prepare champions	In current project trainee Psychiatrist/ Psychotherapist embedded in project team Could be any of the following professionals within organisation with appropriate training: Psychiatrist Psychologist Mental health nurse OT Social worker	Champions process within clinical teams and health organisation and point of contact for advice/support/service development	Health organisation managers, individual clinicians requiring additional support	Champion should be available to attend strategy meetings within health organisation before and during implementation. ACD supporter/ champion should be available as named point of contact for individuals seeking support with process of making ADM.	Increase acceptability of intervention within health organisation. Work with organisation to improve feasibility e.g. systems for awareness and access in a crisis	Acceptability Adoption Appropriateness Feasibility	Previous research suggests health organisation support is crucial and larger scale strategies to tackle access issues are required. ( Stephenson *et al.*, 2020b)
6	Build a coalition	Members of project interdisciplinary research team	Engagement within mental health trust: Members of executive board Mental Health Law Committee Deputy medical director Junior doctors Relevant frontline clinical multi- disciplinary teams Advocacy team Restrictive practice lead Third sector organisations: Bipolar UK Recovery College Lambeth Carers Hub Bethlem Gallery Service User Advisory Group External professional organisations: Royal College of Psychiatrists Mental Welfare Commission for Scotland NHS Education for Scotland NHSX Department of Health and Social Care Pan London Crisis Care Academic partnerships: Coordinate my Care (Royal Marsden Hospital) AdStac team King’s College London	Build internal and external organisational support and expertise for implementation	Throughout the pilot project		Acceptability Adoption Appropriateness Feasibility	
40	Involve executive boards							
24	Develop academic partnerships							
64	Use advisory boards and workgroups							
52	Promote network weaving							
Train and educate stakeholders								
29	Develop educational materials	Template and guidance co- produced by service users, professionals and family members/ friends	Template and guidance offered to those wishing to make ADM documents. Provides structure for service user to state crisis preferences and record of joint recommendations. Ensures compliance with relevant legal frameworks Offers information and guidance on completing the template and addresses common concerns	Service user, clinicians involved in making the document, family members/friends involved in making the document, future clinicians reviewing the service user in crisis	Template should be offered after initial invitation or at point of request for help making ADM document.	1 template emailed or posted to service user (according to preferences and level of tech access/ literacy)	Acceptability Adoption Appropriateness Feasibility	*‘it’s very well written…there’s some questions about kind* * of relapse indicators, and…treatment options that kind* * of left it quite open but did give some guidance, and * *that was perfect for him.’ (Health professional for service * *user 6 Post document interview)* *‘(the form) it was all right…it was clear..the order was * *also alright’ (Service User 7 Post document interview*)
31	Distribute educational materials	ACD supporter Frontline clinical multi- disciplinary teams	Training for psychiatrists Training provided (in person and/or online) for frontline teams during their regular team meetings. Teams include:	CMHT Psychiatric liaison teams Home Treatment/ Community Crisis Teams	Template and guidance materials distributed Clinicians working with service users to make or use ADM documents	Knowledge about ADM documents and the supporting legal frameworks including upcoming reforms Prior to supporting service users in their team to make and use ADM documents	One 30–60 minute didactic training session complimented by active support/ coaching on individual basis 1 email with guidance documents	Increase acceptability of intervention for clinicians and manage anxiety about mental health ADM. Increase feasibility within their system – learning about any necessary adaptations. Previous research ( Thornicroft *et al.*, 2013) suggests that clinician buy in has been a crucial barrier preventing uptake.
15	Conduct educational meetings							
16	Conduct educational outreach visits							
Engage stakeholders								
41	Involve service users and family members	Health professional Supporter Family member/ friend Service user	Service users and their family members were involved throughout the process of making the ADM document	Service users Health professionals Family members/friends	Family/friends and treating health professional were involved in the process as per service users wishes	Usually 1 family member/friend and 1 treating health professional. Varied according to service user preference	Adoption Acceptability Appropriateness	*‘It was a bit therapeutic, you know, because in their way* * they went through the experiences I went through in* * mental health with me.’ (Service User 7 Post Document* * Interview)* *‘I thought it was helpful, really helpful to have everybody* * like in the same space. (Service User 4 Post Document* * Interview)* *‘it will put me more at ease. So that even if I’m not here…* * Then I know that things will be better in place for her’* * (Friend/family for Service User 2 Baseline Interview)* *I’m glad I’ve done it, I think it’s a good thing, it’s opened* * the dialogue up. It feels like we’ve got a team now, in* * place (Service User 4 Post Document Interview)* *It was good because it’s an all-inclusive meeting. You* * know, they include me as the patient, and my family* * members, and my health care professionals…yeah. So* * it was good, you know. Everybody had an input (Service* * User 7 Post Document Interview)*
	Intervene with service users to enhance uptake and adherence	Health professional providing standard care	Active offer of ADM Health professional actively and repeatedly offering opportunity to make ADM.	Service users with previous experience of compulsory treatment or those at high risk	Offer of ADM may be most acceptable to people who have had more than one crises and who have accepted their diagnosis. Suggested timings: Signpost on discharge from an inpatient admission, or during Home Treatment Team/ community crisis care Active offer on intake to CMHT Active offer at point of discharge from CMHT as tool to summarise learning from spell with team	3–4 offers during care pathway	Adoption	*‘I think it was one of the doctors that mentioned it * *and asked if it was something I’d be interested in, at* * the beginning of my sort of crisis intervention…sort of * *treatment….with the home treatment team…and they * *asked again when I was being discharged,’ (Service User * *2 Baseline Interview)* *‘So someone who is having their first crisis, they might * *not be able to recognise it just yet. Like if they’re just* * coming out of a crisis they might not recognise what the* * triggers were. They might have an idea, but they might* * not fully know because it might be like’ (Service User 10 * *Post Document Interview)* *‘with a different colour skin… filling this out has a* * different emotional load’ (Health Professional for Service* * User 14 Baseline Interview)*

***Evaluative and iterative strategies.*** Informal feedback and solution ideas were collected from the participants and collated in field notes throughout the project. At the end of each cycle a meeting was held with a working group to review progress, feedback and discuss any necessary adaptations. Evaluation took place throughout the process and formally in final in depth interviews.

***Provide interactive assistance.*** Making mental health ADM is a relational, bespoke activity that required time and investment from all parties. Health professionals reflected that this was different from ‘business as usual’ and should not be simply rolled into normal processes e.g. Care Planning Approach (CPA) meetings. Some expressed anxiety about the process of completing a Crisis PACk as it was unfamiliar – particularly navigating the legal meaning of the document. The role of a ‘supporter’ was clarified during project. A service user advisory group (SUAG) were consulted about the appropriate language and stance for someone in this role. They advised that language of support was preferred to reflect the power dynamics and ensure that the process respected the primacy of service user ownership and expertise. Participants suggested supporters could be any mental health professional, a peer supporter or advocate with suitable training but expressed concerns that a person in this role would require commitment, advanced listening, and interpersonal skills to ensure the service user did feel truly supported while engaged in a potentially triggering task. There was a preference, amongst all participants, that, although they highly valued involvement from their treating team, the supporter should be someone independent i.e. not the service user’s care coordinator/usual psychiatrist, to manage the power differentials involved.

The supporter provided a 1:1 drafting session with the service user prior to meeting with health professionals which focussed on eliciting the service user preferences and navigating the administrative aspects of completing the document. This drafting support session was seen as having an important role in managing emotional distress from previous traumatic experiences in mental health services, overcoming ambivalence and navigating power imbalance. Service users reported that it meant they felt prepared for meeting with a health professional as they were clear in their own minds about what they wanted. If wished the supporter would chair meetings with treating health professionals/family members and disseminate the document. The boundary for the supporter role was around providing any clinical advice on the content of the document. For health professionals the supporter would provide materials in advance of meeting and offer a coaching session. Most often this took the form of an email with a sample agenda and a brief video call in advance of meeting with the service user. At baseline several professionals expressed concern about the resource and clinical time that making a Crisis PACk would require. However, after being part of the process most felt it was a valuable exercise and worth the upfront investment in the collaborative effort to make the document.

***Adapt and tailor to context.*** A key adaptation occurred at the start of the pilot project in response to the pandemic and the new urgency to implement ADM this generated within the mental health trust. We had planned to pilot the PACT template which was co-produced as part of a previous research project ( Stephenson *et al.,* 2020b). This had a relatively specialist focus around people with a diagnosis of bipolar who wished to self-bind to treatment. However, the mental health trust advised that they wished to design an ADM template in response to anticipated MHA reform, to reduce inpatient bed use and address concerns about physical health ADM if a service user were to contract covid whilst in hospital. This template would need to have a wider reach than the PACT template. To facilitate this adaptation a working group was formed comprising senior SLAM clinicians and IT specialists plus members of the research team which comprised clinical academics (LS, GO, LR) working within the trust and a lawyer (ARK). The working group also had links with a service user advisory group and experts with lived experience within the Recovery College. The PACT was adapted to the ‘Crisis PACk’. This group met after every PDSA cycle completed, in total 4 times over 12 months. The template was reviewed and updated with a synthesis of participant and working group suggestions.

***Develop stakeholder interrelationships.*** During the project it became clear that a clinical champion role was needed to act as a coordinator for ADM and point of contact for people interested in making a document. As interest in ADM increased the advisory and networking side of this role grew to incorporate consultation for other interested academic researchers and clinical bodies. Coalitions were built with: relevant health trust committees (e.g. Trust executive board, Mental Health Law committee, older adult ADM group), third sector organisations (e.g. Recovery College, Bipolar UK), professional bodies (e.g. Royal College of Psychiatrists), governmental bodies (e.g. Department of Health and Social Care), and other research groups (e.g. Adstac group exploring ADM with people from black backgrounds).

***Train and educate stakeholders.*** The Crisis PACk template and guidance was co-produced within the interdisciplinary research team, service user advisors and participants. The guidance was distributed to participants and members of frontline teams that were visited to deliver educational settings. Teams that worked with service users likely to benefit from making Crisis PACks were contacted and offered a training session at a time of their convenience in person or online. This was well received when packaged as a training session about current and future legal provision for ADM.

Key learning from participants was around simplifying the template and guidance. One set of guidance was produced for service users and another for professionals and it was located within the template inside the relevant box for ease of use. The process of making the document was also enormously simplified. The initial guidance contained 7 steps in a narrative format for making the document. After receiving participant feedback about this and asking for solutions this was altered to a visual aid (available on Crisis PACk template within extended data with 3 steps: draft, discuss, disseminate).

Although the materials and training were appreciated, we found that participants valued in person and tailored advice far more highly than referring to guidance documents. The general thoughts around the template were that it was clear, fit for purpose and not a barrier but that support was still required to complete it. The education and training sessions had an important role in awareness raising but we found that the ‘on the spot’ coaching was far more effective in increasing people’s confidence to complete ADM documents. It seemed that, if participants had educational materials alone, they would be unlikely to make the ADM documents.

***Engage stakeholders in ADM process: service users, family/friends, health professional.*** Service users reported finding the collaborative process of making the document to be a therapeutic activity. Service users were given a choice about who to involve. Usually they chose one family member/friend and one health professional from their treating team. The supporter offered to coordinate meetings with the people the service user wished to include. Every effort was made to overcome barriers to engagement including meeting times and mediums (e.g. online/phone etc). Service users highly valued the opportunity to have health professionals and family members/friends involved in making their Crisis PACk. Family member/friend participants expressed views that it was also beneficial for them to be involved in the process to reduce the stress of future crises and empower them to help manage these more confidently and in line with service user wishes.

Participants emphasised the importance of an active, and repeated, offer of support with ADM made at the right time for them. It would not be sufficient to simply wait for service users to ask to make a document. Learning from the project suggested the following points in the clinical pathway would be appropriate to make offers of engaging with ADM: initial signpost on discharge from an inpatient admission, initial signpost during Home Treatment Team/community crisis care, active offer on intake to CMHT, active offer at point of discharge from CMHT as a tool to summarise learning from spell with team. Participants were unified in thinking that although everyone with experience of detention should be offered the opportunity to make an ADM document only people who have accepted their diagnosis and who have had previous experience of managing a crisis might feel ready to take on this task.

An important topic emerging in the interviews was the experience of service users from Black backgrounds within mental health systems. Multiple service users, family/friends and professional participants identified this group as more likely to experience discrimination, micro-aggressions and trauma during mental health treatment. They felt that this made it even more important for service users from this group to make ADM documents yet they faced additional barriers in the form of the increased emotional load required to make the document as well as lower trust in services. When asked to generate potential solutions to these difficulties participants again emphasised the importance of active offers of culturally sensitive support with ADM to all (to avoid risk of selecting those from demographics who might be assumed as more likely to engage with ADM) and suggested community engagement e.g. liaising with religious groups and third sector organisations to raise awareness and increase acceptability.

***Concerns/unintended consequences.*** Participants used the interviews to express their views about the importance of implementation efforts for ADM and the potential unintended consequences if ACD implementation were not taken seriously or appropriately resourced. Key concerns were that it could become a ‘tick box’ exercise, that was rushed and that future professionals would not access or take documents seriously. Participants felt that the consequences of this could be reduced trust and disengagement.

*‘decisions might be made or suggested about medication that then become embedded, and everybody feels slightly paralysed about that. And they’ve not been really carefully thought through, because somebody can’t come along for half an hour and rush through an advance directive.’ (Health Professional for Service User 14 Baseline Interview)*

*‘I’m glad to have it there. I just hope that it gets looked at and it’s kind of utilised as we want it to be.’ (Service User 3 Post Document Interview)*

## Discussion

### Summary of findings

This paper reports on the co-production of implementation strategies for making mental health ADM which are fit for use with upcoming reforms to the MHA in England and Wales. Of those offered the opportunity to make a Crisis PACk over 60% (23/36) accepted and over 70% (17/23) went on to draft the document. Of the group who started drafting over 75% (13/17) went onto complete their document. This pattern suggests that the largest drop out occurs at the offer stage and once people start drafting their document they are more likely to continue. It may suggest service users can quite accurately predict their commitment to making ADM documents.

Service users were most commonly offered the opportunity to make a document by CMHTs rather than inpatient settings or crisis teams. Our experience on the project was that people were usually still too unwell to make Crisis PACks whilst on an inpatient ward. Health professionals most commonly identified the need for making an ADM document rather than service users themselves or family members/friends. The extra time required for making the document was on average a 38 minute session to draft the document (provided by a supporter) and a 78 minute session (including service user, treating health professional, family member/friend and supporter) to discuss and complete the document. Both of these contacts could be completed satisfactorily remotely during dedicated online meetings.

Key strategies adopted by participants and identified as accessible, appropriate and feasible were: providing interactive assistance in the form of an independent, skilled ‘supporter’, training and education for stakeholders including provision of a structured ADM template and guidance and engagement of stakeholders with active offers of involvement to service users and family members/friends.

### International context

These findings build on a developing body of international literature which has confirmed an aspiration/implementation gap for mental health ADM and outlined barriers to implementation. Hypothesised implementation strategies to address these barriers have included: facilitation of ADM documents ( Ruchlewska *et al.,* 2014; Swanson *et al.,* 2006b), appointing clinical champions ( Zelle *et al.*, 2015), stakeholder outreach meetings ( Lenagh-Glue *et al.*, 2021), training and IT support ( Stephenson *et al.,* 2020b; Zelle *et al.*, 2015). This study adds to this literature in that it: offers a deeper exploration of the reasons why support is so crucial for service users in managing trauma and power differentials, addresses key issues in producing materials and processes for ADM, generates and evaluates multiple co-produced implementation strategies, clarifies stakeholders and organises these co-produced implementation strategies within a recognised framework ready for future studies.

The findings of this study are consistent with the clearest message from the literature so far: that service users require support in creating ADM documents ( Swanson *et al.,* 2006b) ( Ruchlewska *et al.*, 2009). In the international literature on ADM several models which are comparable to the Crisis PACk prototype and utilise support have been developed. In the US facilitated ADM was successfully trialled. This model involved offering in person support to write an ADM document and go through all the official procedures to register. Facilitators were research assistants trained by a psychologist and facilitation sessions lasted 120 minutes. Participants who received this support were significantly more likely to go on to complete an ADM document ( Swanson *et al.,* 2006b).

In Europe and the UK the Joint Crisis Plan (JCP) has been trialled. This involves two sessions; an independent facilitator meets with the service user and their care coordinator to prepare for a second planning meeting with a psychiatrist and family member (if desired). This is an informal document with no ability to self-bind. The multi centre UK trial did not show any significant benefit: the study authors concluded this was largely due to lack of clinical buy in and merging the process for creating a JCP with a Care Programme Approach (CPA) meeting which serves more professional purposes ( Thornicroft *et al.*, 2013). The European trial explored different options for facilitators and concluded that a trained peer supporter could achieve better results than clinicians in support for drafting ADM documents ( Ruchlewska *et al.*, 2014). A recent randomised controlled trial in France also concluded that ADM documents completed by service users with SMI supported by peer workers resulted in significantly fewer compulsory admissions ( Tinland *et al.*, 2022). Interestingly, the New Zealand experience suggested that more service users were able to complete their document with a clinician, the key factor being trust rather than nature of the facilitator’s previous experience ( Lenagh-Glue *et al.*, 2021).

Looking at the Crisis PACk model in this context it is most similar to the Joint Crisis Plan in that it involves a supported two-step process. A distinguishing characteristic of the Crisis PACk model is that the emphasis is on the document ownership belonging primarily to the service user. The initial drafting session is 1:1 with the supporter (without a care coordinator). This offers the maximum chance that the service user can freely express their opinions and receive emotional support and preparation for meeting with a health professional. The ‘discussion’ stage similarly involves a healthcare professional and a family member which can be facilitated by the supporter who, significantly, is not a member of the treating team. Also, the Crisis Pack is a formal document making explicit use of the available legal framework and it supports self-binding. A unique part of the process of making a Crisis PACk compared to other similar models is the use of technology. This was necessary during the pandemic and this pilot has demonstrated the feasibility of completing these documents remotely which may increase time efficiency and accessibility for many service users. However, caution should be taken to ensure that non digital approaches remain viable for those without tech access.

### Limitations

A key limitation impacting generalisability is that we had a relatively small sample size and included only those with bipolar. However, this project relied on in-depth qualitative feedback around sensitive topics. All had experience of detention and compulsory treatment under MHA and reported traumatic experiences in this context. This suggests the sample is a ‘hard to reach’ population and representative of those whom ACDs are aimed at. Working consistently with the participants over time allowed the researcher to build relationships with them and supported them to be candid in their feedback. As such the sample size was appropriate and lays a foundation for later work scaling up these strategies. We focussed on service users with bipolar as the most straightforward case of fluctuating capacity to pilot the concept. Future work will be required with service users who have more diverse diagnoses. This paper focusses only on the process of making ADM documents rather than accessing them and applying them in crisis, a future publication will detail the outcomes experienced by participants during the follow up period. A key issue is likely to be around accessibility and IT infrastructure to facilitate this which we did not tackle in this stage of the project.

### Recommendations for further research and ACD implementation

On 10/05/2022 the Queens Speech announced (for the second time) the intention that draft legislation to reform the MHA in England and Wales will be published. In the White Paper which preceded it, a commitment to introducing ACDs as part of these reforms had been made. We do not at the time of writing have the detail of precisely what shape any statutory reform to implement ACDs will take, but the key conclusion of our research is that, whatever shape it takes, NHS mental health trusts should start preparing for ACDs now. Without these preparations it is unlikely that ACDs will achieve their potential to increase service user autonomy, improve outcomes and reduce coercion. The preparations we recommend are set below.

***Extending pilot projects.*** Pilots in other trusts are urgently required so that the implementation strategies proposed here can be scaled up and adapted. We suggest one model to pilot for wider roll out would be an ACD ‘workshop’. This could comprise a multi-disciplinary team made up of a psychiatrist, systemic psychologist, and ACD supporters. This workshop could offer a hub of expertise around facilitating collaborative ACDs, trouble shooting, training and outreach to community groups and third sector organisations.

***Resource for supporters.*** Resources should be made available for independent ‘ACD supporters’ to be trained and work with a range of service users and clinical teams in each health trust. Supporters could come from diverse backgrounds e.g. professional/lived/advocacy/chaplaincy expertise/third sector.

***Resource for training and education for stakeholders.*** Key professional groups (e.g. psychiatrists/social workers/care coordinators/psychologists/mental health nurses) should have mandatory training on making and using ACDs. This should include training around supporting service users to overcome common barriers (e.g. cultural sensitivity, digital poverty, literacy issues, trauma) and managing concerns around mental capacity assessment when making ACDs and applying in crisis.

***Resource for engagement/outreach.*** Health trusts should embed making active offers for support making ACDs at the following points within standard care pathways: initial signpost on discharge from an inpatient admission, initial signpost during Home Treatment Team/community crisis care, active offer on intake to CMHT, active offer at point of discharge from CMHT. These offers should include outreach to family members/friends as per service user preference.

***Building stakeholder interrelationships.*** ACD champions should be appointed to collate expertise and facilitate intra- and inter- agency learning in organisations involved in responding to mental health crises. These organisations include: professional bodies for psychiatrists, nursing, social workers, psychologists, paramedics, emergency physicians and police plus mental health trusts and third sector organisations. Champions should be encouraged to form a national network to build expertise and establish best practice as ACDs are rolled out.

***Evaluative and iterative strategies.*** ACD uptake and use must be monitored to identify inequalities in take up and areas where more support is required. We recommend the following key areas to measure:

- Number ACDs offered
- Number of ACDs created
- Demographics of those creating ACDs (matched to local population)
- Number of ACDs accessed in crisis
- Number of ACDs overridden

## Data availability

### Underlying data

Due to risk of de-anonymisation, the raw underlying qualitative data has not been made freely available. If researchers or referees would like access to the data for re-analysis, please contact the corresponding author (L.A.S.) by email at lucy.a.stephenson@kcl.ac.uk. Applicants who wish to access the data will need to demonstrate that they have a position at a recognised academic institution and secure data storage facilities. Partial data will be made available in response to specific research questions. In accordance with our study protocol data sharing will be proportionate, task-orientated and will occur subject to strict understandings about confidentiality.

### Extended data

figshare: My Crisis PACk.docx. https://doi.org/10.6084/m9.figshare.20072168.v1 (Stephenson, 2022a)

This project contains the template file for the Crisis PACk used in this research.

figshare: Crisis PACk Guidance. https://doi.org/10.6084/m9.figshare.20072195.v1 (Stephenson, 2022b)

This project contains the guidance documents for those making Crisis PACks.

Data are available under the terms of the Creative Commons Attribution 4.0 International license (CC-BY 4.0).
