# Supplementary material for: Preparing for Mental Health Act reform: Pilot study of co-produced implementation strategies for Advance Choice Documents
Source: Wellcome Open Res. 2022 Jul 7;7:182. [Version 1] doi: 10.12688/wellcomeopenres.17947.1 (PMC9433915; doi:10.12688/wellcomeopenres.17947.1)
Supplement: Peer review history

# Peer review: Reviewer response for version 1

**DOI:** 10.21956/wellcomeopenres.19890.r51409

**Published:** 2022-08-31

**Authors:** Colin McKay

**Affiliations:** 1 Centre for Mental Health and Capacity Law, Edinburgh Napier University, Edinburgh, UK

The article discusses the use of improvement methodology to improve the uptake and quality of Advance Choice Documents in relation to mental health care for people with bipolar disorder. It is clearly set out with tables showing how the documents and the process for making them evolved through the improvement process. The study design is appropriate to support the practical, real world implementation of an agreed policy goal. It builds on existing studies and can be used to inform, as recommended, future pilot work in preparation for reforms to the Mental Health Act in England and Wales. The source data is only available on request because of anonymisation, but this should not affect the value of the research.

 This is a useful and timely piece of research. As well as MHA reform in England and Wales, the topic has wider international relevance given the imperative in Article 12 of the UN Convention on the Rights of Persons with Disabilities for 'support in exercising legal capacity', which the UN Committee has confirmed includes Advance Statements ( General Comment Number 1): ‘All persons with disabilities have the right to engage in advance planning and should be given the opportunity to do so on an equal basis with others.’

 In the English context, the usefulness of this work is enhanced by the fact that the UK Government originally proposed a standard format of Advance Choice Document but now proposes a more flexible approach and that ‘it should be led by what the service user feels is most important to facilitating their recovery’ Reforming the Mental Health Act: government response.

 The research highlights that commitment from and support for staff may be as important as supporting the maker of the document. Further work may need to address how time and resources can be found for this in pressured clinical settings.

 The education level of participants seemed relatively high (9 out of 14 university educated). Further work might usefully consider whether different support may be needed for people with different educational backgrounds – the Mental Welfare Commission report on Advance Statements in Scotland found that people from more deprived areas were more susceptible to their Advance Statement being overridden.

 Another possible limitation is that the work focused on supporting people who were already committed to making an advance statement. It is less clear whether this would address the barriers experienced by those who did not choose to make a document – more than half the original cohort. Further research/pilot work may be needed to identify ways to make ADM more attractive to wider group.

 The research suggests that the process works best for those who are accepting of their diagnosis. It would be interesting to follow up on how to support people who have a more fundamental difference of perspective from professionals – who in some ways would be people who might seem most in need of an opportunity to make advance choice

 The research used templates originally based on the Mental Capacity Act, and the design of the packs were changed to cover physical conditions. This suggests that MHA treatment is not the only driver of advance choice making, and future work could consider how to bring together the two different legal frameworks in one advance planning process. Previous research has found barriers to advance directives in the MCA for people with bipolar disorder – see Richard Morriss, Mohan Mudigonda, Peter Bartlett, Arun Chopra & Steven Jones (2020) National survey and analysis of barriers to the utilisation of the 2005 mental capacity act by people with bipolar disorder in England and Wales, Journal of Mental Health, 29:2, 131-138, DOI: 10.1080/09638237.2017.1340613. ^1^

Is the work clearly and accurately presented and does it cite the current literature?

Yes

If applicable, is the statistical analysis and its interpretation appropriate?

Not applicable

Are all the source data underlying the results available to ensure full reproducibility?

Partly

Is the study design appropriate and is the work technically sound?

Yes

Are the conclusions drawn adequately supported by the results?

Yes

Are sufficient details of methods and analysis provided to allow replication by others?

Yes

Reviewer Expertise:

Mental health and capacity law, human rights, medical ethics

I confirm that I have read this submission and believe that I have an appropriate level of expertise to confirm that it is of an acceptable scientific standard.

## References

[1] : National survey and analysis of barriers to the utilisation of the 2005 mental capacity act by people with bipolar disorder in England and Wales. *J Ment Health* .2020;29(2) : 10.1080/09638237.2017.1340613 131-138 10.1080/09638237.2017.1340613 28644738

# Peer review: Reviewer response for version 1

**DOI:** 10.21956/wellcomeopenres.19890.r51408

**Published:** 2022-07-18

**Authors:** Adam Searby

**Affiliations:** 1 School of Nursing & Midwifery, Institute for Health Transformation, Deakin University, Geelong, VIC, Australia

Thank you for the opportunity to review this paper. Generally, I found it well written and adding an interesting implementation/co-design angle to the research around advance directives. My two (minor) suggestions are to:

- Include an explainer on implementation methods used in the background/introduction to ensure the paper is approachable by readers without a background in implementation science.
- Include the most recent data on advance directives in the 'international context' section, as there have been studies completed after 2006/2009.

Thank you for exploring this important area of mental health research.

Is the work clearly and accurately presented and does it cite the current literature?

Yes

If applicable, is the statistical analysis and its interpretation appropriate?

Yes

Are all the source data underlying the results available to ensure full reproducibility?

No

Is the study design appropriate and is the work technically sound?

Yes

Are the conclusions drawn adequately supported by the results?

Yes

Are sufficient details of methods and analysis provided to allow replication by others?

Yes

Reviewer Expertise:

Alcohol and other drug (AOD)/addiction nursing, nursing workforce, mental health nursing

I confirm that I have read this submission and believe that I have an appropriate level of expertise to confirm that it is of an acceptable scientific standard.
